# Preparation of a solid dispersion of Euptox A and evaluation of its biological activity and safety

**DOI:** 10.1371/journal.pone.0320743

**Published:** 2025-03-27

**Authors:** Haojun Li, Runa Zhao, Wei Zhou, Zhili Li, Wenlong Chen

**Affiliations:** Provincial Key Laboratory for Agricultural Pest Management of Mountainous Region, Institute of Entomology, Guizhou University, Guiyang, China; University of California Riverside, UNITED STATES OF AMERICA

## Abstract

Terrestrial mollusks are common agricultural pests that often infest crops for a long time during the growth stage and create holes or notches in vegetable leaves, especially seedlings and young leaves. In this study, a solid dispersion (SD) of Euptox A and polyethylene glycol-4000 was prepared via the melting method. The drug-to-carrier ratio, melting temperature, and cooling temperature of Euptox A SD were optimized. Euptox A SD was tested against *Bradybaena ravida*, *Limax maximus*, and *Oncomelania hupensis* for 48 and 72 h. The toxicity of Euptox A SD was equivalent to that of 10%Meta, a traditional chemical softener. Moreover, the rate of apoptosis in human liver cells (L02) caused by Euptox A SD was low. The toxicity of Euptox A SD to *Pheretima tschiliensis* was low, and the normal development of *Brassica campestris* was not affected. These results indicate that Euptox A SD can be used as an alternative molluscicidal.

## 1. Introduction

*Ageratina adenophora*, also known as *Eupatorium adenophorum*, is a perennial plant and a globally invasive weed. This species is difficult to control, and resources are needed for its prevention and control in recent years [1]. *A. adenophora* can synthesize bioactive chemical components owing to properties such as allelopathy, toxicity, and stress resistance [2–4]. A lactone produced by *A. adenophora* stimulates the mucous membranes of animals and insects and acts as an animal or insect antifeedant [5]. The ciliated seeds and pollen of *A. adenophora* can cause asthma in equines, and in severe cases, lung tissue necrosis and death [6]. Chemical solvent extracts from the flowers and leaves of *A. adenophora* have been shown to effectively kill the third instar larvae of *Culex quinquefasciatus* [7]. One example is 9β-hydroxy-ageraphorone isolated from the petroleum ether extract of *A. adenophora* that has higher acaricidal activity than fenvalerate [8]. Owing to the presence of monoterpenes, sesquiterpenes, and phytosteroids, such as *β*-pinene, limonene, thymol, p-cymene, cymene lactone, caffeic acid, ferulic acid, and dandelion sterol, *A. adenophora* exhibits antibacterial, anti-inflammatory, and antiviral activities [9–11].

*Bradybaena ravida*, *Limax maximus*, and *Oncomelania hupensis* are key harmful mollusks in Chinese agriculture with a wide range of distribution and many host species; both adults and larvae can harm crops [12]. In particular, these mollusks preferably harm Solanaceae, Cruciferae, and Leguminosae crops [13]. The mucus residue and sticky feces excreted after crawling facilitate breeding of bacteria [14]. After crops are damaged, leaves, stems, and fruits are bitten into notches and holes, which seriously reduce the quality of agricultural products and crop safety [15]. The mollusks are not only harmful to crops but also transmit plant pathogens and parasites from humans, livestock, and wild mammals, posing a threat to human health [16,17]. At present, mollusks are controlled mainly by spraying 10%Meta and other chemical pesticides [18]. Long-term use is extremely susceptible to drug resistance, affecting the prevention and control effect. In addition, the substance will also cause harm to the environment, especially to water pollution [19].

Euptox A is a sesquiterpene in *A. adenophora*. Many studies have shown that sesquiterpenes have a wide range of biological activities [4,20,21], and Euptox A has shown high aphid-killing activity. Biochemical and toxicological experiments also have shown that it had an obvious inhibitory effect on the AchE activity and Na-K-ATPase activity of *Aphis gossypii* [22]. In addition, Euptox A is also highly toxic to livestock. It can not only cause allergic bronchopneumonia in horses but also lead to contact dermatitis in cattle, sheep, and other livestock. Additionally, studies have shown that it caused liver damage in mice [23–25].

A solid dispersion (SD) is a mixture of one or more active ingredients dispersed in the solid state in an inert carrier prepared by melting, solvation, spray-drying, or other methods [26,27]. An SD reduces the particle size to its minimum, increases the absorption area, and improves the bioavailability of drugs by dispersing them in a large number of carriers [28]. Abamectin B2 chitosan microspheres prepared by SD technology have been studied to control the cucumber root-knot nematode, and promising results have been obtained in field trials [29]. An 18% clothianidin-embedded sustained-release agent was prepared by the melting method with SD technology, and carnauba wax and polyethylene glycol (PEG) were selected as the composite carriers. The embedding rate was > 93%, and the population reduction rate was ≤ 83.2% [30].

In order to reduce the biological invasion of *A.adenophora*, a substitute agent with more environmental protection was prepared. The present study aimed to prepare an Euptox A SD via the melting method to evaluate its biological activity and safety for mollusk control.

## 2. Materials and methods

### 2.1. Source of Euptox A

The Euptox A extract in the Euptox A dispersion prepared in this study was purchased from Jiangxi Baicaoyuan Biotechnology Co., Ltd.

### 2.2. Mollusks

The *B. ravida*, *L. maximus*, and *O. hupensis* used in this study were sourced from the ecological experimental field of Professor Chen at the Institute of Entomology, Guizhou University (latitude: 26°25′39.62″N; longitude: 106°40′5.81″E; 1090 m altitude). Mollusks were reared following a standard method [31]. Briefly, *B. ravida, L. maximus, and O. hupensis* species were reared in 9 ×  6-cm (diameter ×  length) plastic containers containing 50 g of damp culture soil (120° for 1 h), with a mixture of fresh vegetables and protein-rich feed mass (3:1) for feeding. The top of the container was covered with a plastic film with small holes for ventilation. The environmental conditions were maintained at 25 °C ±  1 °C, 70% ±  5% relative humidity, and a 16:8 h light:dark cycle.

### 2.3. Phytochemical analysis of the *A. adenophora* extract

The melting point of PEG-4000 is 70 °C [32]. To ensure the stability of Euptox A in the melting process, the thermal stability of PEG-4000 was determined at 50 °C–90 °C [33]. After accurately weighing 0.5 g of Euptox A and drying at a constant temperature for 12 h, the content change of Euptox A was measured. The drying temperatures selected were 50 °C, 70 °C, and 90 °C. The content change of Euptox A at these three temperature points was determined within ± 0.13%. The results showed that a melting temperature of 50 °C–90 °C did not affect the stability of Euptox A.

### 2.4. Preparation of Euptox A SD and determination of its dissolution

PEG-4000 was used as the SD carrier. Euptox A was weighed, and PEG-4000 was heated to achieve melting at 70 °C. Euptox A was added to melt at different mass ratios (1:5, 1:10, 1:15, 1:20, 1:25, 1:30, 1:35, and 1:40), stirred continuously until completely melted, cooled and solidified immediately in a refrigerator, kept standing for 3 days to make it brittle, dried in an oven at 30 °C for 24 h, and finally sieved through an 80-mesh sieve to obtain Euptox A SD.

According to the paddle method in Appendix XX C of the China Pharmacopoeia 2010 Edition (Volume II) [34], the release medium comprised a solution of water and 0.3% sodium dodecyl sulfate (SDS), the rotation speed was 100 rpm, and the temperature was 37 °C. After adding Euptox A SD into the dissolution cup, 5-ml samples were taken at 5, 15, 30, 45, 60, and 120 min (the same volume of medium was added to the cup after each sample removal). After filtration through a 0.45-μm microporous membrane (Guangdong Huankai Microbiology Technology Co., Ltd. Mixed Cellulose (CN-CA) Filter), the absorbance was measured at 270 nm, and the cumulative dissolution percentage was calculated to obtain the optimal drug–carrier ratio.

### 2.5. Effect of the melting temperature and cooling temperature on the dissolution

According to the optimal drug–carrier ratio, three different melting temperatures of 65 °C, 70 °C, and 75 °C were selected, and the cooling temperature was set at 0 °C. Euptox A SDs were prepared at different melting temperatures, and dissolution was performed to determine the optimal melting temperature. According to the optimal drug–carrier ratio and melting temperature results, cooling temperatures of −20 °C, 0 °C, and 20 °C were selected, and dissolution was performed using the abovementioned method to determine the optimal cooling temperature.

### 2.6. Bioactivity evaluation of Euptox A SD

Sixty-day-old *B. ravida*, *L. maximus*, and *O. hupensis* were treated using the leaf membrane method with Euptox A SD diluted to 18.75, 37.5, 75, 150, and 300 mg/L in distilled water. Distilled water was used as the negative control, with 10%Meta as the positive control [35]. Each 15 units was a replicate for 3 times. Mortality data were recorded after 48 and 72 h of treatment. The concentration at which 50% of the mollusks died (LC_50_) and the 95% confidence interval (CI) of the LC_50_ were calculated.

### 2.7. Evaluation of the safety and activity of Euptox A SD

Flow cytometry was performed to analyze the degree of apoptosis caused by the Euptox A SD in human normal liver cells (L02), and the acute toxicity of Euptox A SD against *Pheretima tschiliensis* was determined according to the artificial soil method in OECD207 [36]. Euptox A SD was applied to *B. campestris* at the fourth leaf stage, and six plants were treated each time, repeated five times. The plant-height growth rate was calculated after 14 days, and the discoloration, necrosis, deformity, and wilting of *B. campestris* plants were recorded at 1, 7, and 14 days after treatment.

### 2.8. Statistical analysis

IBM SPSS Statistics v.25 software (IBM Corp, Armonk, NY) was used to perform probit analysis to calculate the LC_50_ values. IBM SPSS Statistics v.25 was also used to analyze the dissolution data, one-way ANOVA was performed to assess differences between treatments, and the Least Significant Difference method was used to determine significance. The mortality of mollusks and of *P. tschiliensis* exposed to the LC_50_ was compared by performing the Kruskal–Wallis and Student–Newman Keuls methods (*p* <  0.05) in BioEstat version 5.0 software [37,38]. Flow cytometry fluorescence plots were analyzed using FCS Express 7 software [39]. Origin v. 2021 software was used to plot the curves.

## 3. Results

### 3.1. Phytochemical analysis of the *A. adenophora* extract

The changes in the Euptox A content within ± 0.13% at the three drying temperatures of 50 °C, 70 °C, and 90 °C are shown in Table 1. Melting temperatures between 50 °C and 90 °C did not affect the stability of Euptox A.

**Table 1:** Effect of three temperature points on the thermal stability of 9-ketoeupolyphin.

Temperature (°C)	Total (g)	Content change (g)
50	0.5	0.5 ± 4.3 × 10^ − 4^
70	0.5	0.5 ± 5.2 × 10^ − 4^
90	0.5	0.5 ± 6.4 × 10^ − 4^

### 3.2. Preparation of the Euptox A SD

**Fig 1:**
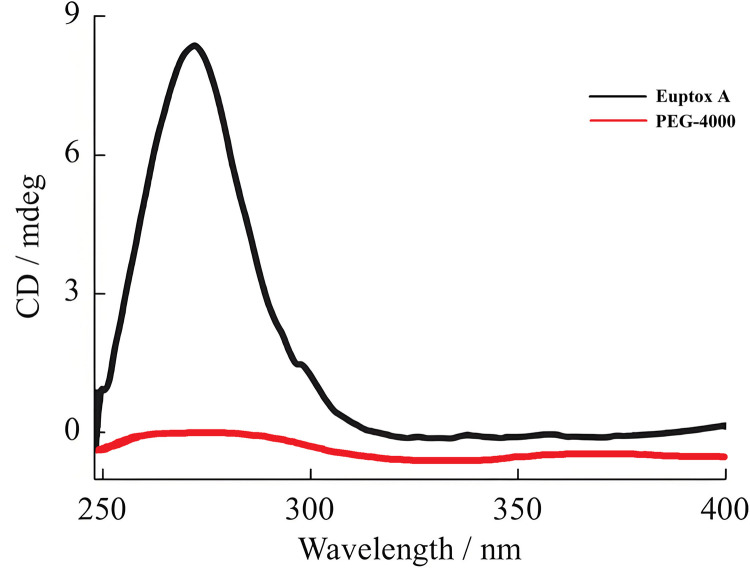
Spectroscopic determination of Euptox A and PEG-4000 at wavelength of 200-400 nm.

Euptox A and PEG-4000 were prepared in an ethanol solution at a concentration of 100 µg/ml, and the absorbance was recorded in the wavelength range of 200–400 nm. The result are shown in Fig 1, Euptox A showed a maximum absorption peak at 270 nm, whereas PEG-4000 showed no absorption between 200 and 400 nm. Therefore, 270 nm was selected as the wavelength for the detection of Euptox A. Euptox A was diluted to make a series of solutions with mass concentration of 1, 5, 10, 20, 50μg/ml respectively, and linear regression was carried out with mass concentration of Euptox A (c, μg/ml) as abscissa and peak area (A) as ordinate, and the regression equation was A = 28.82c-4.26 (r = 0.9999, n = 5).

Briefly, a certain amount of PEG-4000 was accurately weighed and placed into a small beaker. The sample was heated to achieve melting (at 65 °C), and a small amount of Euptox A was added to prepare different mass ratios of Euptox A and PEG-4000 (1:5, 1:10, 1:15, 1:20, 1:25, 1:30, 1:35, and 1:40). Heating was continued until the mixture appeared clear, after which the sample was immediately placed in a refrigerator at 0 °C to cool and solidify. It was further continued to stand until it became brittle and then dried for 3 days in a dryer to obtain Euptox A SD.

**Fig 2:**
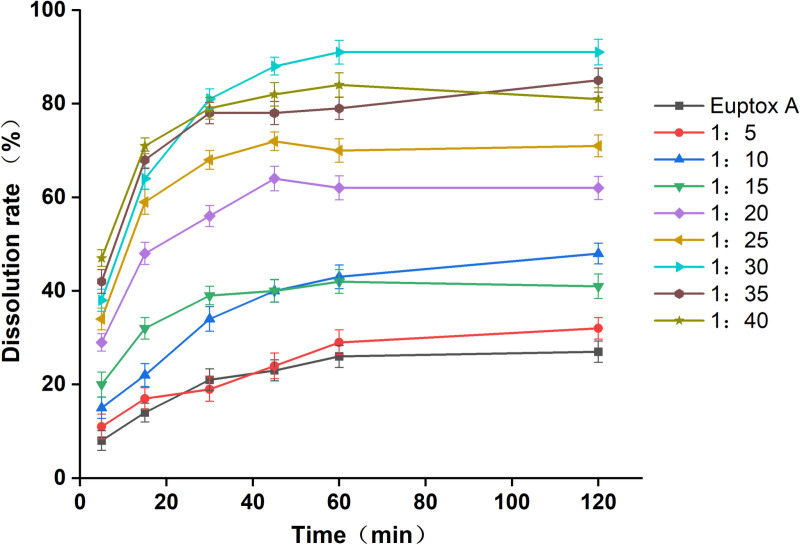
Dissolution versus time profiles of Euptox A solid dispersions at different drug–carrier ratios.

The dissolution profile of the prepared Euptox A SD was determined according to the first method (basket rotating method) of dissolution determination in Appendix XX C of the China Pharmacopoeia (Volume II), Edition 2010, with 900 ml of purified water as the medium, a constant temperature of (37 °C ±  0.5 °C), a rotation speed of 100 rpm, and addition of 0.3% SDS to the dissolution medium. An appropriate amount of the dissolution solution (simultaneous addition of equal volumes of medium) was taken at 15, 30, 45, 60, and 120 min and filtered through a 0.45-μm microporous membrane. The filtrate was used for analysis. Fig 2 shows the SD dissolution results with different drug–carrier ratios at different time points.

As shown in Fig 1, within 120 min, the dissolution of Euptox A SD improved compared with that of the active pharmaceutical ingredient (API). When the ratio of the two ranged between 1:5 and 1:15, the SD dissolution was ≤ 48%. When the ratio of the two was 1:20–1:25, the dissolution was approximately 72%. When the ratio of the two was 1:30, the dissolution was > 90%. When the ratio of the two was 1:35–1:40, the dissolution was approximately 80%.

### 3.3. Effect of melting temperature on the dissolution of Euptox A SD

**Fig 3:**
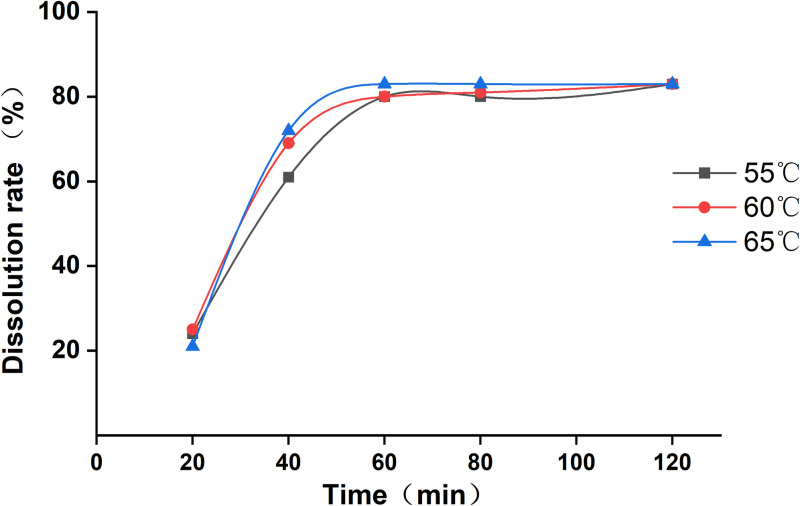
Dissolution profiles of Euptox A SD at different melting temperatures.

To test the effect of melting temperature on the dissolution of Euptox A SD, we confirmed that the drug-loading ratio was 1:30; the dissolution of Euptox A SD was ideal; the SD at a drug–carrier ratio of 1:30 was weighed; the test melting temperatures were set to 55 °C, 60 °C, and 65 °C; the cooling temperature was set to 0 °C; and the SDs for testing at different melting temperatures were prepared. The dissolution curves of the Euptox A SD at different melting temperatures are shown in Fig 3.

### 3.4. Effect of the cooling temperature on the dissolution of Euptox A SD

**Fig 4:**
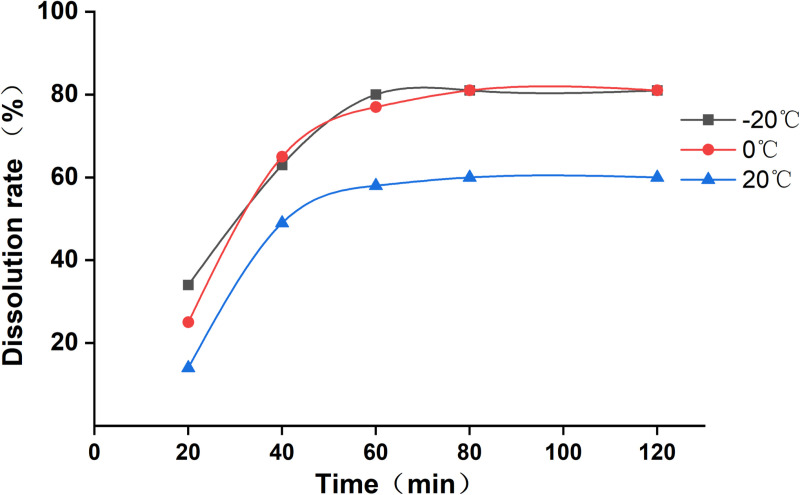
Dissolution curves of Euptox A SD at different cooling temperatures.

To test the effect of cooling temperature on the dissolution of Euptox A SD, samples with a drug:carrier ratio of 1:30 were weighed, the melting temperature was 60 °C, the cooling temperatures were − 20 °C, 0 °C, and 20 °C, and different SDs for testing were prepared. When the cooling temperature was lower, i.e., the cooling rate was faster, the particle size of the amorphous particles or crystals was smaller, and the dissolution rate was faster, but the overall dissolution rate was not obviously improved. Therefore, a cooling temperature 0 °C was selected. The dissolution curves of Euptox A SD at different cooling temperatures are shown in Fig 4.

### 3.5. Bioactivity evaluation of Euptox A SD

The extract of Euptox A SD and 10%Meta were diluted to five gradient concentrations, and 45 samples were selected for each concentration. Subsequently. After 7 days, the number of samples fatalities was recorded. The results of the toxicological test are presented in Table 2. Using statistical data from IBM SPSS Statistics 25 software and Probit for analysis to calculate LC_50_, results are shown in Table 3.

**Table 2:** Determination of toxicity of Euptox A SD and 10%Meta to *B. ravida, L. maximus* and *O. hupensis.*

Object	Reagent Concentration (mg/mL)	Slug number	Euptox A SD		10%Meta	
**48h Deaths**	**72h Deaths**	**48h Deaths**	**72h Deaths**
*B. ravida*	18.75	45	9	11	14	14
	37.5	45	15	18	18	18
	75	45	28	30	27	28
	150	45	33	36	34	35
	300	45	36	40	39	40
*L. maximus*	18.75	45	9	11	12	12
	37.5	45	17	19	20	20
	75	45	25	26	25	26
	150	45	30	32	29	30
	300	45	36	37	37	38
*O. hupensis*	18.75	45	14	16	16	16
	37.5	45	19	21	20	21
	75	45	27	29	29	29
	150	45	32	33	33	34
	300	45	35	37	37	37

**Table 3:** Determination of the biological activity of Euptox A SD against *Bradybaena ravida*, *Limax maximus,* and *Oncomelania hupensis.*

Reagent	Mollusks	Time (h)	LC_50_ (mg/L)	95% CI (mg/L)	Regression equation	X^2^
Euptox A SD	*B. ravida*	48	93.72	77.79–113.89	Y = 2.44x + 1.82	0.83
		72	61.82	54.01–79.84	Y = 2.3x + 4.17	1.78
	*L. maximus*	48	102.72	83.79–128.31	Y = 2.15x + 4.26	0.76
		72	80.03	71.78–108.95	Y = 2.12x + 4.12	0.88
	*O. hupensis*	48	72.58	57.46–97.83	Y = 2.31x + 4.52	1.02
		72	49.35	43.21–70.96	Y = 2.35x + 3.98	0.85
10%Meta	*B. ravida*	48	67.61	53.94–84.28	Y = 2.15x + 3.62	0.13
		72	63.36	48.25–79.34	Y = 1.94x + 3.53	3.08
	*L. maximus*	48	92.44	84.21–126.46	Y = 2.12x + 4.2	5.63
		72	86.60	69.97–101.38	Y = 2.27x + 4.57	0.91
	*O. hupensis*	48	67.42	49.36–73.84	Y = 2.58x + 4.39	2.64
		72	55.63	41.47–82.48	Y = 2.55x + 4.21	3.12

The results showed in Table 3 that the Euptox A SD had a toxic effect on the adults of *B. ravida*, *L. maximus*, and *O. hupensis*, and the toxicities of Euptox A SD and 10%Meta to *B. ravida*, *L. maximus*, and *O. hupensis* were similar. When the exposure time was 48 h, the LC_50_ values to *B. ravida* and *L. maximus* were higher for Euptox A SD than for 10%Meta, and the LC_50_ values of Euptox A SD and 10%Meta to *O. hupensis* were equivalent, indicating that the toxicities to *B. ravida* and *L. maximus* were lower for Euptox A SD than for 10%Meta, whereas the toxicities to *O. hupensis* were equivalent. When the exposure time was 72 h, the LC_50_ values to *B. ravida*, *L. maximus*, and *O. hupensis* were lower for Euptox A SD than for 10%Meta, which indicated that the toxicities against *B. ravida*, *L. maximus*, and *O. hupensis* were higher for Euptox A SD than for 10%Meta.

### 3.6. Evaluation of the safety and activity of Euptox A SD

**Fig 5:**
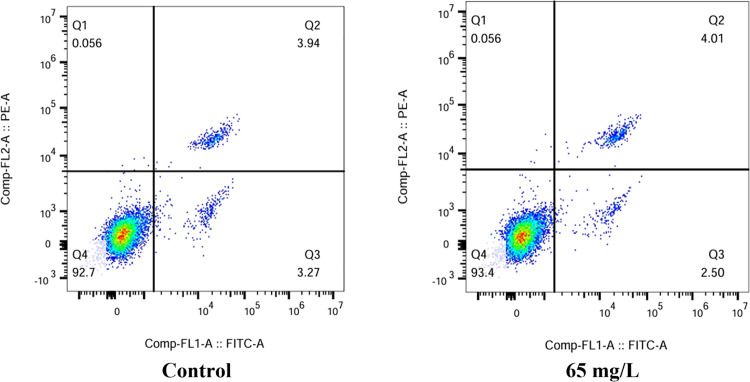
Ratio of apoptotic cells to necrotic cells after exposure to Euptox A SD using flow cytometry (Q1: viable cells; Q2 and Q3: apoptotic cells; Q4: necrotic cells).

To assess the risk of pesticides to humans, experiments using normal human hepatocytes (L02) were conducted. Fig 5 shows that the Euptox A SD may cause cell damage to varying degrees. The total apoptosis rate of L02 cells in the control and treated groups were 3.27% and 2.50%, respectively. The proportion of viable cells in the treated and control groups was similar. The experimental results showed that the Euptox A SD was less toxic to L02 cells.

The Euptox A SD easily entered soil during mollusk control, so it and may cause damage to *Pheretima tschiliensis*. To evaluate the safety of Euptox A SD, its toxicity to *P. tschiliensis* was tested, as shown in Table 4. After 7 days, the LC_50_ of *P. tschiliensis* was 34.58 mg/kg (soil), and after 14 days, the LC_50_ of *P. tschiliensis* was 31.46 mg/kg (soil). These results showed that the LC_50_ of the Euptox A SD was > 10.0 mg/kg (soil) and the toxicity level to *P. tschiliensis* was low.

**Table 4:** Determination of the toxicity of Euptox A SD to *Pheretima tschiliensis.*

Time	LC_50_ (mg/kg) (soil)	Regression equation	Virulence rating
7 d	34.58	Y = 5.12x − 2.18	Low
14 d	31.46	Y = 4.05x − 3.68	Low

Evaluating the effects of pesticide formulations on plants is an important factor in formulation development. The application of Euptox A SD may affect the growth of *B. campestris* seedlings, and its effect on the height growth rate of *B. campestris* seedlings is shown in Table 5. Compared with the blank treatment, there was no significant difference in the height growth rate of the *B. campestris* seedlings treated with different concentrations of Euptox A SD. Therefore, the Euptox A SD prepared in this study was found to be safe for *B. campestris* and could be used for mollusk control.

**Table 5:** Effect of Euptox A SD on the height growth rate of *Brassica campestris.*

Treatment concentration (mg/L)	Height growth rate
0	4.52 ± 0.36a
40	4.58 ± 0.43a
50	4.51 ± 0.25a
60	4.56 ± 0.54a
70	4.53 ± 0.41a
80	4.50 ± 0.44a

Same lowercase letters indicate no significant difference (*p* >  0.05).

## 4. Discussion

In recent years, many active pesticide ingredients have encountered serious obstacles to practical application due to their low water solubility. Approximately 30%–40% of all drugs have low water solubility [40]. Poor water solubility seriously hinders the release of active ingredients in drugs and ultimately limits their field application [41]. Many techniques have reportedly improved the solubility and dissolution rate of these poorly soluble drugs. These techniques include the following: 1) increasing the surface area by reducing the particle size; 2) forming water-soluble complexes; 3) solubilizing with surfactants; and 4) using amorphous SD technology to form amorphous and solid solutions to reduce crystallinity [42,43]. Among these technologies, SD technology is the most easily industrialized technology and is currently also used as a classic strategy to increase the solubility and dissolution rate of poorly soluble drugs.

In our study, PEG-4000, a water-soluble polymer, was selected as the carrier, and Euptox A with a higher melting point was melted at a certain temperature due to its low melting point. Guo et al. prepared a clothianidin SD via the melting method. Carnauba wax and PEG were used as composite carriers. When preparing multi-component mixed solid carriers, the melting and uniform dispersion of active ingredients in the carrier should be considered to improve the persistence of the SD. The field efficacy test showed that the persistence period against citrus psyllid was ≤ 3 months, and the control effect was 25% higher than that at the same dose 96 days after application [30]. Yu et al. successfully prepared an abamectin B2 SD using the complex coacervation method. Abamectin B2 was dissolved in an organic solvent and mixed evenly into the oil phase by adding an emulsifier. A 22% abamectin B2 SD with an irregular appearance was successfully prepared. The cumulative release rate in the soil for 80 days was 74.7% (soil moisture content >  15%, soil temperature >  20 °C) [29]. There are various SD preparation methods, and an increasing number of pesticides are being prepared as SDs.

The Euptox A SD prepared in this study showed toxic effects on *B. ravida*, *L. maximus*, and *O. hupensis*, whereas 10%Meta was a moderately toxic agent, which exerted rapid effects on target objects within a short time [44]. However, its control time was short and the effect remained unsustainable [45,46], which was consistent with the biological activity determined in this study. The LC_50_ values of Euptox A SD against *B. ravida* and *L. maximus* were higher than those of 10%Meta exposed for 48 h. However, after 48 h, the LC_50_ values of Euptox A SD against *B. ravida* and *L. maximus* were lower those of 10%Meta exposed for 72 h, indicating that the toxicity of Euptox A SD was higher than that of 10%Meta. The toxicities of Euptox A SD and 10%Meta against *O. hupensis* were equivalent at exposure times of 48 and 72 h. These results are consistent with those of some botanical insecticides, showing that the efficacy was relatively slow and the toxicity gradually increased and persisted for a longer period [47–49].

The advantages of SD technology are that it is a simple process with good reproducibility, but the solubility of many compounds is low. Therefore, it should be used for solubilizing drugs at low doses [50]. A sustained-release SD formulation using a polymer carrier has been successfully prepared and serves as an example of SD technology in pesticide formulation. However, it must be continuously enhanced to improve the sustained-release performance of pesticides in certain applications. For example, SDs with different particle sizes and distinct appearance shapes are needed to produce differentiated SDs [51,52]. Furthermore, SDs with different environmental responsiveness can be prepared by using the targeted release characteristics of SDs. It is also possible to use carriers with different characteristics to prepare SDs that are sensitive to pH, temperature, humidity, and light. Drugs in SDs mainly exist in molecular, amorphous, or microcrystalline forms [53,54].

Compared with synthetic agents, plant-derived softeners are less stable and readily decompose under the influence of environmental factors, such as light, temperature, and air [55]. Once the phytochemicals are extracted, their components may be affected by oxidative damage, chemical transformation, or polymerization reactions [56]. In addition, their quality may deteriorate further over time, and plant extracts may lose attributes, such as odor, taste, color, and concentration [57]. Plant-derived softeners are not suitable for use where long-term residual effects are required due to the diversity and instability of their components [58]. To solve this problem, it is necessary to construct a suitable drug-delivery system to enhance the application value of plant-active substances.

In this study, considering the cost factor, we used melting method as the preparation method, and PEG-4000 as the drug carrier. If we can change the preparation method and use the composite carrier, whether there will be better effect is our future research direction. The cooling step in the preparation process and the time of transferring the drug to the refrigerator may affect the effect of the drug. In the future, we will continue to improve the preparation process of drugs and select more perfect preparation methods that conform to drug properties.

## 5. Conclusions

Euptox A is a type of sesquiterpene substance in *A. adenophora*. Euptox A and PEG-4000 were combined to prepare Euptox A SD via the melting method. The drug-loading ratio, melting temperature, and cooling temperature in the preparation process were optimized. The obtained Euptox A SD showed toxic effects against *B. ravida*, *L. maximus*, and *O. hupensis*, and the toxicity was equivalent to that of 10%Meta. In the biosafety assessment of nontarget organisms, the apoptosis rate of L02 cells in the Euptox A group was low, the toxicity against *P. tschiliensis* was low, and no adverse effect on the growth of *B. campestris* was detected. These results indicate that the melting method can be used to prepare a 9-keto-eupolyphin SD with excellent properties that is green, effective, and safe.
